# Managing Pulmonary Embolism With Right Ventricular Thrombus in In Vitro Fertilization-Related Pregnancy: A Clinical Insight

**DOI:** 10.7759/cureus.51383

**Published:** 2023-12-31

**Authors:** Saleh A Ba-shammakh, Eman A Al-zughali, Nashaat A Al-Shami, Ali H Al-Darobi, Hammam A Abuaisha, Shadi Karabsheh

**Affiliations:** 1 General Surgery, Princess Basma Teaching Hospital, Irbid, JOR; 2 Internal Medicine, The Islamic Hospital, Amman, JOR; 3 Banner MD Anderson Cancer Center, Banner Gateway Medical Center, Arizona, USA

**Keywords:** maternal-fetal health, pregnancy, ivf-related thrombosis, in vitro fertilization ivf, pulmonary embolism

## Abstract

This case study provides an insightful examination of the management of high-risk pulmonary embolism (PE) in a 27-year-old pregnant patient following in vitro fertilization (IVF). Overlapping symptoms of PE and typical pregnancy manifestations, coupled with concerns about radiation exposure from diagnostic imaging, presented unique diagnostic challenges. Despite the heightened risk of thrombosis during pregnancy and elevated D-dimer levels, a conservative approach was strategically employed. This involved therapeutic anticoagulation using low-molecular-weight heparin, leading to significant patient improvement without the need for invasive interventions. This case highlights the imperative for a judicious yet proactive approach in managing PE among pregnant patients, meticulously considering both maternal and fetal health risks.

## Introduction

Pulmonary embolism (PE) is recognized as a primary cause of maternal mortality in developed nations, with mortality rates ranging between 11 and 15 per million births, notably in regions like Europe and the United States [1-2]. In the United Kingdom, PE, as an integral part of venous thromboembolism, is responsible for a third of maternal deaths [2]. This elevated mortality rate is often associated with delayed diagnosis and inadequate thromboprophylaxis [2]. D-dimer levels inherently increase during pregnancy, necessitating careful interpretation in conjunction with other diagnostic assessments [3-4]. Definitive diagnosis typically relies on imaging methods such as high-probability V/Q scans or contrast-enhanced imaging, often supported by echocardiography [5-6]. Addressing intracardiac thrombi, particularly in the right ventricle, presents challenges due to the limited availability of evidence-based guidelines [7]. Treatment modalities range from anticoagulation therapy to surgical interventions [8]. This case exemplifies the success of conservative management in a pregnant patient with high-risk PE.

## Case presentation

A 27-year-old female patient, in her eighth week of pregnancy with a history of gestational hypothyroidism and two prior unsuccessful attempts at in vitro fertilization (IVF) due to male factors, achieved her first pregnancy through IVF induction. She is a non-smoker and has no history of substance abuse.

The patient presented at the emergency room (ER), complaining of persistent retrosternal chest pain, which had progressively worsened over the past seven days. The pain exacerbated during exertion and deep inspiration and was alleviated partially by rest. This discomfort led to shortness of breath, even during minimal activity. Additionally, she noticed heart palpitations, often coinciding with the onset of chest pain. Importantly, she denied any lower limb swelling at the time of admission. Two weeks prior to her presentation, she had sought medical attention in the emergency department due to right lower limb swelling. However, a previous right lower Doppler ultrasound showed no signs of acute DVT.

During the physical examination at the ER, the patient appeared comfortable, with a chest examination showing good bilateral air entry and no added sounds. However, her oxygen saturation was recorded at 89% during this ER visit. Her other vital signs at admission were: blood pressure (BP) 106/75 mmHg, heart rate (HR) 120 beats per minute, respiratory rate (RR) 20 breaths per minute, and temperature (T) 36.8°C. Due to the decreased oxygen saturation, she was administered supplemental oxygen via a nasal cannula at a flow rate of 2-3 liters per minute. Upon transfer to the Critical Care Unit (CCU), her oxygen saturation improved to 97%.

**Figure 1:**
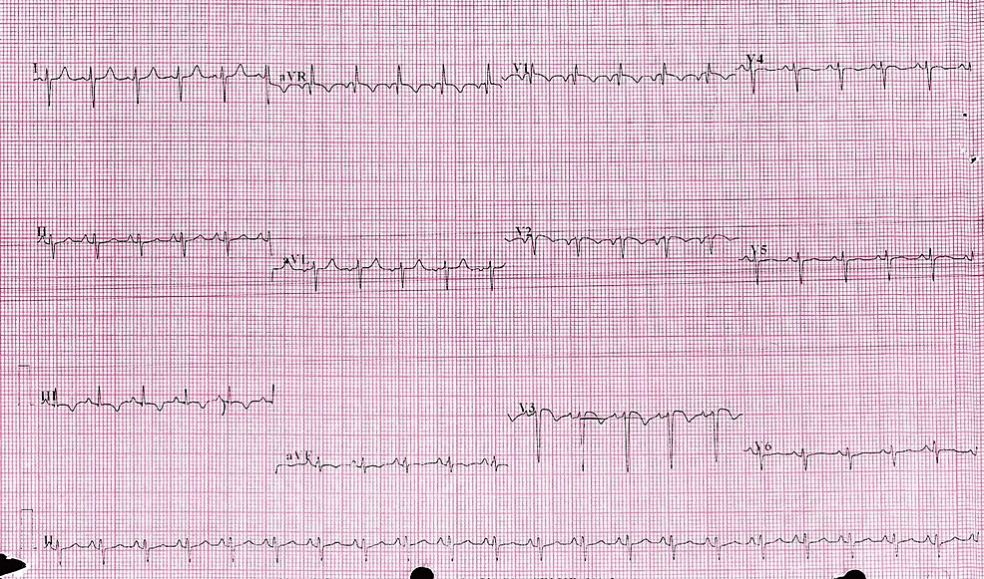
Electrocardiogram (ECG) Findings The ECG demonstrates sinus tachycardia, an S1Q3T3 pattern, right-axis deviation, QR pattern in V1, T-wave inversion from V1 through V3, and incomplete right bundle branch block.

Among her laboratory findings, the only notable abnormality was an elevated D-dimer/HS level at 6.6 mg/L (normal range <0.5 mg/L). Other parameters such as troponin I, creatine kinase (CK), prothrombin time (PT), partial thromboplastin time (PTT), international normalized ratio (INR), complete blood count (CBC), kidney function tests (KFT), liver function tests (LFT), and urine analysis were within normal ranges. Her thrombophilia profile, including tests such as cardiolipin antibody IgG, IgM, beta-2 glycoprotein antibody IgG, IgM, lupus anticoagulant, antinuclear antibody (ANA), factor II mutation (G20210A), factor V Leiden mutation (G1691A), MTHFR gene mutation (C677T), protein C, protein S/free, homocysteine in blood, factor VIII: C, and antithrombin activity, were negative. Notably, a chest X-ray was not performed due to the early stage of the patient's pregnancy and concerns about potential radiation exposure. The electrocardiogram indicated sinus tachycardia, S1Q3T3, T-wave inversion from V1 through V3, right-axis deviation, and incomplete right bundle branch heart block (Figure 1).

**Figure 2:**
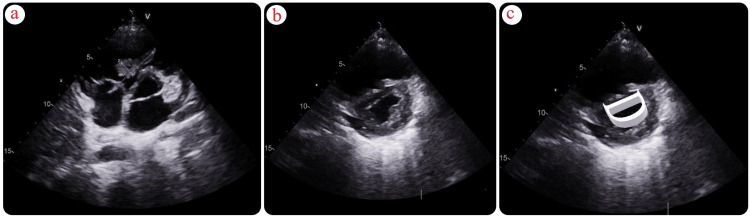
Transthoracic Echocardiogram: Right Ventricular Findings (a) Transthoracic echocardiogram (parasternal, short-axis view, aortic valve level) demonstrating in-transit thromboemboli in the right atrium (RA) crossing the tricuspid valve (TV) into the right ventricle (RV). (b,c) Transthoracic echocardiogram (parasternal short-axis view at midventricular level) demonstrating right ventricle (RV) dilatation and interventricular septal flattening (D-shape).

A transthoracic echocardiogram revealed a dilated right ventricle (RV) with a base measuring 4.6 cm (normal <4.1 cm), displaying hypokinesis of the RV free wall with sparing of the apex, and a tricuspid annular plane systolic excursion (TAPSE) of 1.4 mm (normal >16 mm). A long hyperechoic mass, attached to the tricuspid valve chordae tendineae and protruding into the right ventricle, measured approximately 5 x 0.8 cm, suggestive of a thrombus in transit (Figure 2). The left ventricle was normal in size, displaying normal systolic function, with an ejection fraction of 55% (normal range 50-70%). It had a D-shaped configuration and paradoxical septal movement. There was +2 to +3 tricuspid regurgitation with a maximal pressure gradient (PGmax) of 45 mmHg (normal <30 mmHg), trace posterior effusion, and a dilated inferior vena cava (IVC) measuring 2.2 cm (normal 1.2-1.7 cm) with respiratory phasic loss. The estimated pulmonary artery systolic pressure was 60 mmHg (normal ≤35 mmHg).

**Figure 3:**
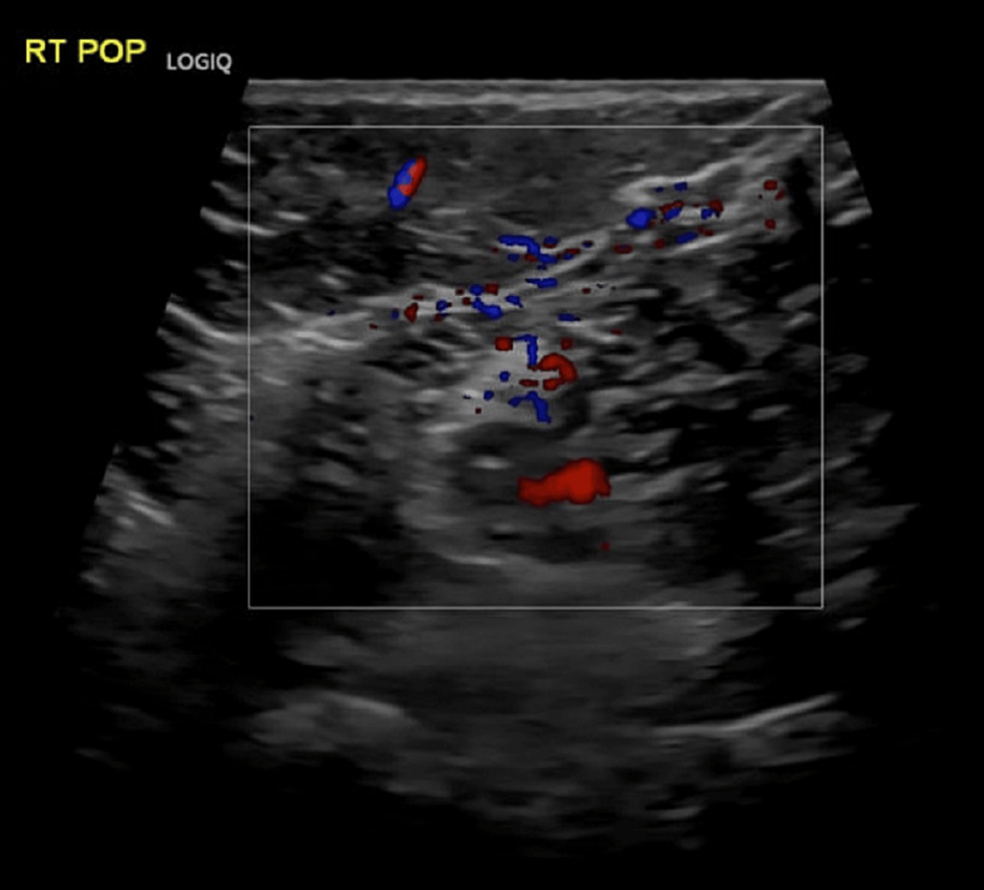
Doppler Ultrasound of the Right Popliteal Vein Ultrasound shows acute and subacute deep vein thrombosis in the right popliteal vein.

The patient was transferred to the CCU for close monitoring and initiated on therapeutic enoxaparin (60 mg subcutaneously twice daily). Additionally, she was prescribed digoxin (0.125 mg orally once daily) to improve her right ventricular function. Other medications included thyroxine, folic acid, ondansetron, aspirin, esomeprazole, and dydrogesterone. Due to persistently declining oxygen levels, the patient was placed on a nasal cannula at a flow rate of 2-3 liters per minute. A bilateral Doppler ultrasound revealed a segment of the right popliteal vein to be dilated, non-compressible, and displaying internal echogenic material, indicative of acute DVT. Another segment showed partial dilation, partial compressibility, and internal echogenic material along with partial recanalization, indicative of subacute DVT (Figure 3).

**Figure 4:**
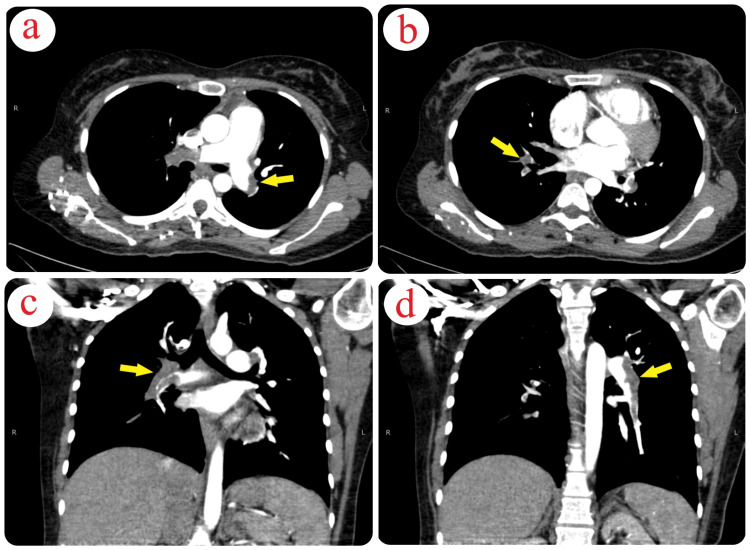
CT Angiogram Indicating Pulmonary Embolism (a-d) The CT angiogram displays filling defects in the pulmonary arteries, consistent with a significant pulmonary embolism.

The patient and her husband were consulted regarding a CT angiogram to assess the extent of the suspected pulmonary embolism, which would guide further management. The CT angiogram revealed filling defects in the main right and left pulmonary arteries, their lobar branches, and right segmental branches, suggesting a major embolism (Figure 4).

On day two, a follow-up echocardiogram showed the thrombus still visible with +1 tricuspid regurgitation, a pulmonary artery systolic pressure of 55 mmHg (normal ≤35 mmHg), an IVC measuring 2.2 cm (normal 1.2-1.7 cm), and a collapsed wall.

An interventional radiologist was consulted for possible intervention, but the size and position of the thrombus made the procedure risky. The decision was made to consider surgical intervention versus thrombolysis. The cardiothoracic surgery team was on standby for potential surgery. The patient and her family discussed treatment options, including continuing with enoxaparin throughout the pregnancy, considering thrombolysis, or opting for surgery to remove the thrombus with the insertion of an IVC filter. The obstetrics team also participated in the discussions, addressing the risks of thrombolysis and expressing support if it was deemed necessary to save the patient's life. The patient and her husband chose to continue with enoxaparin in the coming days, with a plan to pursue surgery if she became hypotensive and hemodynamically unstable. The thrombophilia workup results were negative.

**Figure 5:**
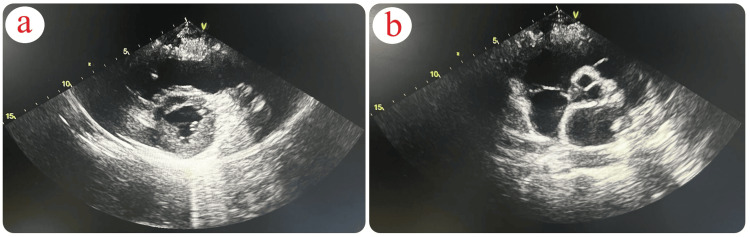
Normal Echocardiogram on ICU Day 4 (a) Transthoracic echocardiogram (parasternal short-axis view) showing the restoration of normal right ventricular size. (b) Transthoracic echocardiogram (parasternal short-axis view, at aortic valve level) showing the resolution of the thrombus.

On the fourth day of her intensive care unit stay, the patient's family decided to proceed with surgery due to her borderline blood pressure, which was fluctuating between 90-105 mmHg systolic and 50-70 mmHg diastolic pressure. Before surgery, another echocardiogram was performed for follow-up, unexpectedly revealing a normal-sized right ventricle with a base measuring 4.1 cm, normal systolic function, trace tricuspid regurgitation with a maximal pressure gradient (PGmax) of 25 mmHg (normal <30 mmHg), no thrombus in the right ventricle or right atrium, a normal-sized left ventricle, normal ejection fraction, trace posterior effusion, a normal-sized IVC, and a reduced pulmonary artery systolic pressure of 30 mmHg (normal ≤35 mmHg) (Figure 5).

Her vital signs remained within normal ranges, and the patient was subsequently transferred to the ward for observation. During her stay, she demonstrated the ability to walk normal distances without experiencing shortness of breath. She no longer required nasal oxygen and was on room air with oxygen saturation levels around 98%. The patient was ultimately discharged with a prescription for enoxaparin (60 mg subcutaneously every 12 hours) and was advised to continue this medication throughout her pregnancy until delivery.

One week after discharge, the patient returned for a follow-up appointment. A repeat echocardiogram confirmed the previous findings, showing a stable and normal cardiac condition. Additionally, she was counseled on maintaining a daily physical exercise routine for at least 30 minutes and on the importance of wearing compression stockings for her legs to reduce edema and improve venous return. She was advised to be vigilant about any changes in her symptoms and to seek immediate medical attention if she noticed any concerning signs, especially those indicative of a potential thrombotic event. This comprehensive care plan was designed to ensure her ongoing well-being, the health of her pregnancy, and to mitigate the risk of future thrombotic complications.

## Discussion

In expectant mothers, PE symptoms often resemble those encountered in non-pregnant individuals, complicating the diagnosis. This is further complicated as common pregnancy symptoms, like shortness of breath experienced by approximately 70% of pregnant women, are also indicative of a normal pregnancy and often stabilize as the pregnancy advances [9-12].

Alterations in hemostasis during pregnancy, marked by elevated levels of various coagulation factors, amplify thrombosis risks, rendering pregnant women more prone to blood clots [13-15]. Data from Sweden and Denmark demonstrate an increased incidence of venous thromboembolisms and PEs in the initial trimester of IVF pregnancies [16-18].

PE-induced hypotension primarily stems from reduced cardiac output and stroke volume, exacerbated by heightened pulmonary vascular resistance (PVR). This increase in resistance is due to pulmonary vessel blood clots and hypoxic vasoconstriction within the pulmonary arterial system [13-14]. Elevated PVR hinders right ventricular blood flow, causing right ventricular enlargement and intraventricular septum distortion. This distortion subsequently impairs left ventricle filling and diminishes cardiac pump efficiency [13-14].

For imaging, the potential hazards of ionizing radiation from CT scans, particularly teratogenicity and oncogenicity, are more pronounced in fetal exposure [19]. This necessitates a judicious selection of imaging modalities during pregnancy. Research, primarily involving animal studies, has established a threshold of exposure necessary to initiate teratogenic effects [19]. While common procedures such as chest X-rays, V/Q scans, and chest CTs have relatively low radiation doses, minimizing exposure remains a critical concern [19].

In response to these concerns, the American Thoracic Society recommends a conservative strategy that prioritizes non-invasive and radiation-free techniques for initial diagnostics [19]. For pregnant patients with lower extremity symptoms, compression ultrasound (CUS) is advocated as the primary imaging modality, owing to its favorable safety profile [19]. In instances where CUS results are inconclusive, and further diagnostic clarity is essential, a chest X-ray is advised as the subsequent investigative step [19]. Based on CXR results and clinical judgment, clinicians decide between a V/Q scan or CT Pulmonary Angiography (CTPA), also considering the accessibility of these techniques [19].

CTPA, often chosen for its rapid availability, is weighed against ventilation-perfusion SPECT, an alternative that offers reduced radiation exposure [20]. This consideration is particularly salient for younger patients concerned about radiation implications for lung and breast tissue [20]. It is crucial to recognize the variable accuracy of CT scans, which can sometimes lead to false positives [20]. Although concerns about fetal exposure to ionizing radiation are often amplified beyond the actual risks [21], the need for timely and accurate diagnosis and management should not be understated [21]. The consequences of delayed or missed diagnoses can be more harmful than the potential radiation risks [21].

In certain contexts, especially in populations with a low incidence of PE, CT imaging may become the preferred method [2]. Its widespread availability and diagnostic comprehensiveness, capable of offering alternative diagnoses, render it invaluable [19]. Nevertheless, the inherent trade-off in terms of radiation exposure warrants careful consideration [19].

In pregnancies, thrombolysis, typically advised against, may become essential in severe PE cases [22]. Considering PE's significant role in maternal mortality, early detection and meticulous monitoring are imperative [21-22]. Managing PE in patients in early pregnancy or with a history of unsuccessful pregnancies poses challenges, necessitating a delicate balance between maternal well-being and pregnancy preservation. The impact of medical interventions in such scenarios is often gradual yet profound [21-22].

The optimal strategy for treating right heart thrombus (RHT) in PE patients remains ambiguous. Treatment options vary from anticoagulation to more invasive procedures like systemic thrombolysis, mechanical thrombectomy, and surgical embolectomy [23]. Research, including Barrios et al.'s work and other studies, yields mixed outcomes, with no clear preference for systemic thrombolysis over anticoagulation [24]. However, some retrospective analyses indicate higher mortality rates with sole anticoagulation compared to thrombolytic or surgical methods [10-11].

Therapeutic anticoagulation using low-molecular-weight heparin or unfractionated heparin is deemed safe during pregnancy, with minimal maternal and fetal bleeding risks and no known teratogenic effects [25]. Although limited trial data exist, thrombolysis has shown efficacy without significant fetal bleeding or teratogenic risks, though maternal hemorrhage remains a concern [25]. Surgical thrombectomy, involving cardiopulmonary bypass, significantly increases the risk of fetal loss [26]. Therefore, a comprehensive, multidisciplinary approach, emphasizing collaborative decision-making, is essential for optimal treatment in these intricate cases.

A 2002 retrospective study analyzing 177 cases of right heart thromboembolism, as reported by Rose et al., based on treatment methods, found thrombolytic therapy more effective than anticoagulation or surgical approaches, underscoring thrombolytics' efficacy [27].

However, this case study, while insightful, has limitations. Firstly, PE diagnosis in our patient was challenging due to symptom overlap with typical pregnancy signs, possibly causing initial diagnosis delays. This highlights the importance of heightened PE awareness in pregnant patients, even with ambiguous symptoms. Secondly, while a CT angiogram was eventually utilized for assessing PE and cardiac anatomical landmarks to guide further management, the initial avoidance of certain imaging techniques due to pregnancy-related radiation concerns might have impacted the timeliness and accuracy of the treatment plan. This underscores the complexity of balancing maternal-fetal safety with the need for definitive diagnostic and interventional radiological landmarks. Lastly, although conservative management proved successful in this instance, its applicability may not be universal. The unique nature of each IVF-related pregnancy necessitates tailored treatment plans, and our approach may have limitations in varying clinical contexts.

## Conclusions

Managing high-risk PE in IVF-related pregnancies presents unique challenges, such as symptom overlap with pregnancy and concerns about imaging methods. This case, despite its limitations, highlights the effectiveness of conservative treatment using low-molecular-weight heparin, underscoring the need for a balanced approach that prioritizes the safety of both the mother and fetus. Although it contributes valuable insights into PE management during pregnancy, emphasizing the importance of individualized care and advocating for ongoing research in this specialized field, it also underscores the need for more extensive research to confirm the efficacy and safety of conservative management strategies in this specialized patient population.

## References

[1] James AH, Jamison MG, Brancazio LR, Myers ER (2006). Venous thromboembolism during pregnancy and the postpartum period: incidence, risk factors, and mortality. Am J Obstet Gynecol.

[2] Bowyer L (2008). The Confidential Enquiry into Maternal and Child Health (CEMACH). Saving mothers’ lives: reviewing maternal deaths to make motherhood safer 2003-2005. The seventh report of the confidential enquiries into maternal deaths in the UK. Obstet Med.

[3] (2023). Thromboprophylaxis during pregnancy, labour and after vaginal delivery. Royal College of Obstetricians and Gynaecologists.,Guideline no. 37.

[4] Nijkeuter M, Huisman MV (2006). Diagnosing pulmonary embolism in pregnancy: is there a role for D-dimer as a stand-alone test?. Crit Care Med.

[5] (2023). Pulmonary embolism in pregnancy: clinical presentation and diagnosis. https://www.uptodate.com/contents/pulmonary-embolism-in-pregnancy-clinical-presentation-and-diagnosis?search=pulmonary%20embolism%20in%20pregnancy&source=search_result&selectedTitle=1~150&usage_type=default&display_rank=1#H1312795.

[6] Ba-Shammakh SA, Al Jayyousi OA, Abu-Hussein M, Abokhsab MM, Al-Thnaibat MH, Haj-Freej HM, Al-Bourah AM (2023). Bilateral deep vein thrombosis (DVT) as a harbinger of lung adenocarcinoma: a rare presentation. Cureus.

[7] Patel M, Wei X, Weigel K, Gertz ZM, Kron J, Robinson AA, Trankle CR (2021). Diagnosis and treatment of intracardiac thrombus. J Cardiovasc Pharmacol.

[8] Lai E, Alishetti S, Wong JM, Delic L, Egrie G, Rosenblatt A (2019). Right ventricular thrombus in transit: raising the stakes in the management of pulmonary embolism. CASE (Phila).

[9] Marik PE, Plante LA (2008). Venous thromboembolic disease and pregnancy. N Engl J Med.

[10] (1990). Value of the ventilation/perfusion scan in acute pulmonary embolism. Results of the prospective investigation of pulmonary embolism diagnosis (PIOPED). JAMA.

[11] Goldhaber SZ (1998). Pulmonary embolism. N Engl J Med.

[12] Weinberger SE, Weiss ST, Cohen WR, Weiss JW, Johnson TS (1980). Pregnancy and the lung. Am Rev Respir Dis.

[13] (2023). Overview of acute pulmonary embolism in adults. T., Kabrhel, C., Mandel, J., & Finlay, G. (2022.

[14] Prisco D, Ciuti G, Falciani M (2009). Hemostatic changes in normal pregnancy. Hematol Meeting Rep.

[15] Ba-Shammakh SA, Al-Zughali EA, Kalaji ZH, Al-Bourah AM, Al-Shami NA (2023). Clinical dilemmas in immune thrombocytopenic purpura with diffuse alveolar hemorrhage: diagnosis, treatment, and outcomes. Cureus.

[16] Henriksson P, Westerlund E, Wallén H, Brandt L, Hovatta O, Ekbom A (2013). Incidence of pulmonary and venous thromboembolism in pregnancies after in vitro fertilisation: cross sectional study. BMJ.

[17] Rova K, Passmark H, Lindqvist PG (2012). Venous thromboembolism in relation to in vitro fertilization: an approach to determining the incidence and increase in risk in successful cycles. Fertil Steril.

[18] Hansen AT, Kesmodel US, Juul S, Hvas AM (2014). Increased venous thrombosis incidence in pregnancies after in vitro fertilization. Hum Reprod.

[19] Dado CD, Levinson AT, Bourjeily G (2018). Pregnancy and pulmonary embolism. Clin Chest Med.

[20] Miller WT Jr, Marinari LA, Barbosa E Jr (2015). Small pulmonary artery defects are not reliable indicators of pulmonary embolism. Ann Am Thorac Soc.

[21] (2023). Diagnostic imaging in pregnant and lactating patients. Kruskal, J. B., Levine, D., & Chakrabarti, A. (2023.

[22] (2023). Approach to thrombolytic (fibrinolytic) therapy in acute pulmonary embolism: Patient selection and administration.. Rivera-Lebron, B., Weinberg, A. S., Mandel, J.

[23] Torbicki A, Galié N, Covezzoli A, Rossi E, De Rosa M, Goldhaber SZ (2003). Right heart thrombi in pulmonary embolism: results from the International Cooperative Pulmonary Embolism Registry. J Am Coll Cardiol.

[24] Barrios D, Rosa-Salazar V, Jiménez D (2016). Right heart thrombi in pulmonary embolism. Eur Respir J.

[25] Fuller KP, Turner G, Polavarapu S, Prabulos AM (2013). Guidelines for use of anticoagulation in pregnancy. Clin Lab Med.

[26] Jha N, Jha AK, Chand Chauhan R, Chauhan NS (2018). Maternal and fetal outcome after cardiac operations during pregnancy: a meta-analysis. Ann Thorac Surg.

[27] Rose PS, Punjabi NM, Pearse DB (2002). Treatment of right heart thromboemboli. Chest.

